# Digital storytelling as a method in health research: a systematic review protocol

**DOI:** 10.1186/s13643-018-0704-y

**Published:** 2018-03-05

**Authors:** Kendra L. Rieger, Christina H. West, Amanda Kenny, Rishma Chooniedass, Lisa Demczuk, Kim M. Mitchell, Joanne Chateau, Shannon D. Scott

**Affiliations:** 1 College of Nursing, Rady Faculty of Health Sciences, University of Manitoba, 317 Helen Glass Ctr, 89 Curry Pl, Winnipeg, MB R3T 2N2 Canada; 2 La Trobe Rural Health School, LaTrobe University, Bendigo, Australia; 3 Max Rady College of Medicine, University of Manitoba, Winnipeg, Canada; 4 Elizabeth Dafoe Library, University of Manitoba, Winnipeg, Canada; 5 Red River College, Department of Nursing, School of Health Sciences and Community Services, Winnipeg, Canada; 6 Faculty of Nursing, University of Alberta, Edmonton, Canada

**Keywords:** Digital storytelling, Stories, Arts, Arts-based, Visual research methods, Systematic review, Ethical considerations, Health research

## Abstract

**Background:**

Digital storytelling is an arts-based research method with potential to elucidate complex narratives in a compelling manner, increase participant engagement, and enhance the meaning of research findings. This method involves the creation of a 3- to 5-min video that integrates multimedia materials including photos, participant voices, drawings, and music. Given the significant potential of digital storytelling to meaningfully capture and share participants’ lived experiences, a systematic review of its use in healthcare research is crucial to develop an in-depth understanding of how researchers have used this method, with an aim to refine and further inform future iterations of its use.

**Methods:**

We aim to identify and synthesize evidence on the use, impact, and ethical considerations of using digital storytelling in health research. The review questions are as follows: (1) What is known about the purpose, definition, use (processes), and contexts of digital storytelling as part of the research process in health research? (2) What impact does digital storytelling have upon the research process, knowledge development, and healthcare practice? (3) What are the key ethical considerations when using digital storytelling within qualitative, quantitative, and mixed method research studies? Key databases and the grey literature will be searched from 1990 to the present for qualitative, quantitative, and mixed methods studies that utilized digital storytelling as part of the research process. Two independent reviewers will screen and critically appraise relevant articles with established quality appraisal tools. We will extract narrative data from all studies with a standardized data extraction form and conduct a thematic analysis of the data. To facilitate innovative dissemination through social media, we will develop a visual infographic and three digital stories to illustrate the review findings, as well as methodological and ethical implications.

**Discussion:**

In collaboration with national and international experts in digital storytelling, we will synthesize key evidence about digital storytelling that is critical to the development of methodological and ethical expertise about arts-based research methods. We will also develop recommendations for incorporating digital storytelling in a meaningful and ethical manner into the research process.

**Systematic review registration:**

PROSPERO registry number CRD42017068002.

**Electronic supplementary material:**

The online version of this article (10.1186/s13643-018-0704-y) contains supplementary material, which is available to authorized users.

## Background

Digital storytelling is an arts-based research method that has the potential to meaningfully capture participants’ lived experiences and share research findings in a highly engaging manner [1, 2]. The method involves the creation of 3- to 5-min visual narratives that “synthesize images, video, audio..., and text to create compelling accounts of experiences” [3], p. 186. This innovative research method holds potential to elucidate complex stories in a compelling and accessible manner and increase participants’ and users’ engagement with the research process. Additionally, arts-based knowledge translation approaches, such as digital storytelling, can elicit nuanced meaning that may otherwise be unreachable for diverse audiences [4].

In the current context of rapidly changing digital technology, researchers must keep pace with novel research approaches that hold significant potential to engage users (i.e. patients, family members) as well as other members of healthcare and research communities. A highly technological world is rapidly changing the definition of what it means to be an informed and contemporary researcher [5]. This digital revolution has ushered in a multiliterate age in which humans engage and communicate through various modalities [6]. Increasingly, people are documenting their lives on social media, whether that be communicating a personal achievement, sharing life experiences, or conveying what it is like to live with illness. Face-to-face contact and virtual presence are becoming equally important as meaningful avenues for expressing lived experiences. Email, Instagram™, Facebook™, and Twitter™ are some of the digital mediums that now connect people more frequently than landlines and regular mail. There have been significant advancements in technology over the last two decades that have shaped the digital revolution. Smart phones and tablets are essentially pocket-sized personal computers. ‘Apps’ shape photos and videos into engaging, personalized media content. It is critical that researchers find innovative approaches to respond to these societal shifts.

Drawing on a growing body of interdisciplinary research from the field of neuroscience, Groff [7] proposed a theory of whole-mindedness for understanding the diverse cognitive processing (visual and language-based) that occurs in the human brain. Groff posits that there are three distinct, but interactive cognitive processing systems at play: visual-object (i.e. static images), visual-spatial (i.e. moving images), and verbal (i.e. language-based). She argues that within contemporary society, the proliferation of digital technology has created a visual revolution, and with that, non-verbal processing skills (visual-object and visual-spatial) now hold dominance. Individuals living in diverse global contexts are not only consumers of high-level multimodal, multimedia visual content; they are also actively engaged in creating and sharing it with others [7]. Unfortunately, focused attention and integration of approaches congruent with visual processing systems have not kept pace with current research inquiry methods. Digital storytelling offers researchers a novel medium to address visual processing systems within the research process. It integrates all three of the cognitive processing systems described by Groff [7] and therefore may be a research method that can facilitate a deeper level of self-expression and understanding within diverse areas of inquiry.

Health researchers from diverse areas of specialization (i.e. mental health, oncology, public health) have used digital storytelling [1, 8–12]. It has been utilized for the educational development of healthcare professionals [13, 14] and as a healthcare intervention [15–17]. Health researchers are increasingly employing digital storytelling as a way to harness the communicative power of digital technology and facilitate the creation and sharing of stories with a worldwide audience. This emerging research approach allows participants to express their thoughts and feelings using a familiar platform that facilitates the creation of poignant personal stories [8]. It is argued that the act of communicating experiences, thoughts, and feelings through this medium can empower participants in the context of very challenging life experiences [2, 9]. The participatory nature of digital storytelling facilitates a highly effective approach for promoting participants’ psychosocial health and well-being as well as elucidating rich narrative data and revealing hidden stories [3]. This method may be particularly useful in qualitative health research, by mediating a profound symbolic exploration of the affective and embodied aspects of healthcare experiences [10].

Digital storytelling has significant potential to initiate community dialogue about issues that are pressing and concerning to research participants [3, 18, 19]. For instance, Lenette and colleagues [9] conducted a study using digital storytelling as a method to share the complex challenges faced by single refugee women. Another research team used digital storytelling as a novel method for sexual health promotion with African American youth living in the inner city [20]. It has been used as an arts-based knowledge translation strategy to share participants’ stories with diverse audiences and engage healthcare professionals in critical reflection of their practice [21, 22]. Given digital media's vast and nearly instantaneous impact, the use of digital storytelling as an innovative knowledge translation approach has the potential to significantly decrease the time between knowledge generation and knowledge implementation.

While many advantages of digital storytelling have been proposed, it can bring significant, often unanticipated ethical challenges to the surface [23]. Given the relative currency of this research method, most institutional ethics boards do not have clearly defined processes in place to ensure ethical safeguards are comprehensively addressed. Within the digital storytelling process, participants share images and information about themselves, which often involves the use of their photographs and voices. Digital storytelling may have negative impacts for participants. Understanding these potential consequences will facilitate the development of ethical processes to mitigate unnecessary harms. To date, the systematic development of an appropriate and ethically sensitive process specific to its use within the research process has not occurred. Although ethical guidelines have been put forth for this storytelling modality [3, 24], these guidelines are not based on a systematic review of the research literature or focused on its use in health research. The importance of maintaining participant confidentiality when using digital stories in public health media has been acknowledged [3]. However, there are also ethical questions about removing a participant’s right to choose to have their story, voice, and digital story shared in a public forum. Who decides, and how researchers/research ethics boards should address these ethical issues, needs to be clearly articulated. These ethical dilemmas highlight the urgency of conducting a systematic review to support the development of guidelines for the use of digital storytelling in health research.

To date, two reviews have been completed on digital storytelling and another review is in progress. The first is a scoping review in mental health [1], the second is a literature review for its use with pediatric cancer patients as a form of reflection [25], and the third is a review that is currently underway which aims to examine it as a pedagogical strategy [26]. No knowledge synthesis work has previously analyzed the use of digital storytelling as a method in health research. Given the significant potential for using it within the research process, a critical review and synthesis of its use in health research is urgently needed to facilitate its maturation into a rigorous research method with clearly defined ethical guidelines. As researchers increasingly adopt digital storytelling within health research, it is essential to develop knowledge about the impact, methodology, and ethical processes for guiding its implementation.

### Aim and review questions

The overall aim of this review is to identify and synthesize evidence on the use, impact, and ethical considerations of using digital storytelling in health research. The questions that will guide this review are as follows: (1) What is known about the purpose, definition, use (processes), and contexts of digital storytelling as part of the research process in health research? (2) What impact does digital storytelling have upon the participants, research process, knowledge development, and healthcare practice? (3) What are the key ethical considerations when using digital storytelling within qualitative, quantitative, and mixed method research studies? For this review, the term impact is conceptualized broadly to encompass the influence, understanding, and negotiated meaning that emerges in the process of digital storytelling, as opposed to looking at the effectiveness on predetermined measurable outcomes.

## Methods

In this systematic review, we will synthesize evidence about the use of digital storytelling in health research studies using established systematic review methods [27]. This review is unique in that it has a focus on a research method as opposed to a phenomenon or intervention [28] . Although qualitative, quantitative studies and mixed methods studies will be included, only narrative data related to our research questions will be extracted. The PRISMA-P checklist [29] guided the development of this protocol (see Additional file 1). This review protocol is registered with PROSPERO [30].

### Inclusion and exclusion criteria

#### Study design

We will include quantitative, qualitative, and mixed method study designs. Qualitative studies can include, but will not be limited to, designs such as phenomenology, grounded theory, ethnography, action research, and feminist research. Quantitative studies can include, but will not be limited to, randomized controlled trials, non-randomized controlled trials, quasi-experimental studies, before and after studies, prospective and retrospective cohort studies, case control studies, descriptive cross-sectional studies, and analytical cross-sectional studies for inclusion. Mixed methods studies can include any combination of these qualitative and quantitative approaches. We will consider published and unpublished articles from January 1, 1990, to present to align with the emergent nature of digital storytelling [10]. We will include only English language articles due to the time and the cost of acquiring and translating articles. There remains controversy surrounding the benefit of expending significant resources for translation [31]. If a quantitative or mixed methods study does not report narrative data, it will be excluded from the review.

#### Participants and setting

Study participants will encompass pediatric or adult populations, their families, and/or health care professionals. The context of the studies will be health research, which includes research conducted in healthcare settings (e.g. clinics, hospitals, community outreach, home visits) or by medical, nursing, or allied healthcare professionals. The research can take place in any geographical location.

#### Intervention

The review will include any primary research studies that use digital storytelling as a method at any point in the research process (i.e. recruitment, data collection, data analysis, knowledge translation). Digital storytelling has been defined as a “creative arts process that is used to capture personal stories, using images and sound in a three to five-minute digital clip” [1], p. 183. The reviewers will exclude the article if digital storytelling is used solely as a therapeutic or pedagogical intervention, for example, within a therapeutic process, a therapeutic intervention, or a teaching strategy.

#### Outcomes

We will extract outcomes related to how digital storytelling impacts the research process (e.g. participants’ engagement and role in the research process, ethical considerations and procedures as described by the researchers in the article, researchers’ and participants’ narrative comments or evaluation of using digital storytelling, findings/conclusions of the study that are relevant to the digital storytelling method, and knowledge translation initiatives).

### Literature search strategy

A specialized healthcare librarian will conduct a rigorous search of the literature for potentially eligible studies. Informed by a preliminary literature search for ‘digital storytelling’ and its variants, a MEDLINE database search strategy, reflecting the range of possible terminology to capture studies relevant to digital storytelling will be translated for each additional database to be searched (see Additional file 2). We will use search tools and strategies specific to each database, including truncation of keywords where appropriate, use of thesaurus terms and subject headings, and combining terms and search strings with the appropriate Boolean operators. We will search the following databases and resources: MEDLINE, PsycINFO, Academic Search Complete, CINAHL, Cochrane Central Register of Controlled Trials, Web of Science (incorporating Social Sciences Citation Index and Arts & Humanities Citation Index), Art Full Text, Art Bibliographies Modern, and Google Scholar. We will search the reference lists of identified articles for additional studies, and forward citations of identified articles will be retrieved using the tools available in resources such as Scopus and Google Scholar. We will search for ongoing or recently completed trials in ClinicalTrials.gov and the International Clinical Trials Registry Platform. We will also search for grey literature in the Dissertations & Theses database, in targeted websites, including StoryCenter [32], Patient Voices [33], and Community Story Collective [34], and by using a limited version of the search strategy in Google.

### Study selection

We will export the search results from the databases to an EndNote library where we will identify and remove duplicate citations, and manage all records. We will use a two-step process for screening retrieved articles. Two independent reviewers will screen titles and abstracts against inclusion criteria following the removal of duplicates, and we will label each study as include, exclude, or unsure. The full text for all articles classified as include or unsure will be retrieved. These articles will be examined independently by two reviewers and evaluated as include or exclude using a screening form (see Additional file 3). We will resolve all disagreements through discussion, and if necessary, a third reviewer will adjudicate unresolved differences.

Prior to the title/abstract screening, we will ensure consistency and rigour during the screening process by randomly selecting 10 articles to assess interrater reliability. The articles will be independently reviewed by each team member against inclusion and exclusion criteria. A scorer sheet will be completed and a kappa’s co-efficient calculated to measure agreement and identify any issues with title/abstract screening procedures. We will consider an acceptable kappa co-efficient to be 0.80 or above based on a sample of ten articles. In the case of a lower kappa result, team discussion will occur on differences in scores. Inclusion/exclusion criteria will be clarified and testing of interrater reliability and discussion will be repeated until agreement reaches the substantial level.

### Data extraction

Data extracted will include context, purpose, uses, benefits, challenges, ethical considerations, and procedures of digital storytelling when used in research, and its impact on research processes, products, and healthcare practices. We will pilot a standardized data extraction form created for this review (Additional file 4) with five included studies. We will then revise the data extraction form in consultation with review team members. One reviewer will extract the data, and a second reviewer will check the data extraction. The reviewers will extract the following data from included studies:

1. Title, authors, publication date, journal title
2. Study purpose, design, and methods
3. Context and participants’ characteristics (e.g. clinical or community setting, geographical location, socio-demographic variables, and diagnosis).
4. Description of the digital storytelling purpose, framework, and processes
5. Role and training of the researcher(s) regarding digital storytelling
6. Participants’ engagement and role in the research process
7. Ethical considerations and procedures as described by the researchers in the article
8. Researchers’ and participants’ narrative comments or evaluation of using digital storytelling in health research (i.e. effectiveness, feasibility, usefulness, impact on research study/findings, issues)
9. Findings of the study that are relevant to the digital storytelling method
10. Study conclusions
11. Knowledge translation initiatives (i.e. if and how they used the digital stories in their dissemination of the research findings)

### Quality appraisals

Two independent reviewers will critically appraise included studies using the Quality Assessment Tool for Quantitative Studies [35] and the Joanna Briggs Institute Checklist for Qualitative Research [36] (Additional files 5 and 6). The Quality Assessment Tool for Quantitative Studies [35] assesses studies on the eight criteria: selection bias, study design, confounders, blinding, data collection methods, withdrawals/dropouts, intervention integrity, and data analysis. The Joanna Briggs Institute Checklist for Qualitative Research [36] assesses studies to see if there is congruity between the research methodology and the stated philosophical perspective, research question, methods used to collect data, representation and analysis of data, and interpretation of results, as well as other trustworthiness criteria. Reviewers will receive pre-training, which will include conducting quality appraisals with the tool on another set of articles (*n* = 5). The reviewers will compare results and discuss their differences, which will increase inter-rater reliability with the quality appraisal tools. Any disagreements will be resolved through discussion between the two reviewers, and if needed, through adjudication by a third reviewer.

Articles will not be excluded based on quality appraisal as the focus of this review is on the synthesis of data related to the digital storytelling method as opposed to primary study results. However, the quality appraisal assessments will provide understanding of how digital storytelling has been implemented as a method. For example, if there are 25 included studies in the review, and 15 of them are rated as having low methodological quality, this knowledge can identify issues and inform recommendations to improve the rigour of future studies. Thus, the quality appraisals will be reported and inform the interpretation and discussion of the review findings. This quality appraisal process will also allow us to identify and discuss exemplars of high-quality studies that have used digital storytelling.

### Data analysis and synthesis

We will present the extracted narrative data in an evidence table organized by study design, clinical context, or research process/stage incorporating the digital story. We will synthesize extracted data through a narrative synthesis [27]. This analytical process will involve a preliminary synthesis and an exploration of the relationships that are evident in the data. The heterogeneity of the studies reviewed will be considered in the process of the narrative synthesis. A minimum of two reviewers will independently code the data and conduct a thematic analysis. The reviewers will describe the use, impact, and ethical considerations of digital storytelling in health research by developing descriptive themes to answer each review question. The review team will engage in ongoing, iterative discussions to deepen and extend the initial analysis produced. Given that this systematic review will address how digital storytelling has been employed across the research process, we will not primarily focus on synthesizing the research findings of the included studies, but will analyze the impact of digital storytelling on the research process. This critical analysis will provide insight into why, when, and how digital storytelling impacts research processes [37]. We will also highlight any methodological limitations of the studies that are identified in our quality appraisals of the work. Since we aim to develop methodological and ethical guidelines, it is critical for us to consider the quality of the studies that underpin these guidelines. This process will allow us to develop salient recommendations to improve future research studies using digital storytelling.

### Integrated knowledge translation plan

We will base the dissemination of the systematic review findings on the Canadian Institutes of Health Research guide to knowledge translation [38]. We will use an adapted integrated knowledge translation plan that includes intensive end-of-project dissemination activities. Integrated knowledge translation incorporates knowledge end users throughout the systematic review to facilitate effective uptake of the research findings [38]. National and international researchers who have expertise in using digital storytelling in health research will be consulted during the analysis phase of the systematic review, as well as in the planning of dissemination activities (researchers based in Canada and Australia). The research team will also actively engage three research advisory groups, facilitating feedback from three Canadian research sites.

We will integrate arts-based approaches into the dissemination plan. Specifically, the research team will create three digital stories. The first digital story will focus on the systematic review findings, the second on ethical implications and guidelines for the use of digital storytelling as a research method, and the third will articulate its use as a health research method. This approach will facilitate a creative, active engagement with the research findings rather than relying solely on more passive, traditional knowledge translation approaches [22]. The research team will also share the findings at local/national/international academic conferences and publish a detailed systematic review manuscript of the findings, in addition to derivative methodological papers (i.e. ethical considerations/guidelines). Finally, we will develop a visual infographic that will be used to disseminate the findings widely using traditional and more innovative social media contexts such as Twitter, on blogs, Instagram, and on academic websites. A one-page executive summary outlining specific research recommendations, ethical guidelines and implications for future research will also be developed and shared extensively using social media, and other channels (i.e. Arts Health Network Canada, The Arts Health Early Career Research Network). These diverse avenues of dissemination will ensure the findings are shared broadly with researchers, practitioners, healthcare consumers, and the public.

## Discussion

The inclusion of qualitative, quantitative and mixed method studies conducted in diverse healthcare settings will elucidate a comprehensive understanding of the breadth, usefulness, and limitations of digital storytelling as a method in health research. We will resolve challenges incurred during data extraction and synthesis in this novel review by discussion among members of the research team, which includes national and international researchers with in-depth expertise in both the substantive (digital storytelling) and methodological (systematic review) areas of this review. One limitation of this review is that only studies published in English will be included, and there may be research in other languages that could contribute meaningfully to the findings. The generalizability/transferability of the findings may be limited as a result of this inclusion criterion.

This rigorous systematic review will be crucial to the development of methodological expertise about using digital storytelling as a formal research method. This review will illuminate why and when digital storytelling enhances the research process or dissemination of findings. This work will also provide significant methodological guidance related to ethical considerations when using digital storytelling in health research. The results will inform recommendations for the use of this method within health research, and may have wider applicability for other research fields. We will disseminate the recommendations through accessible mediums to diverse groups of knowledge-users. This review will be essential to informing the future work of researchers employing digital storytelling and will play a pivotal role in building research capacity in arts-based research methods.

## Additional files

Additional file 1: PRISMA-P 2015 Checklist. (DOCX 38 kb)

Additional file 2: MEDLINE (Ovid) Search Strategy. (DOCX 55 kb)

Additional file 3: Verification of Study Eligibility Form. (DOCX 15 kb)

Additional file 4: Standardized Data Extraction Form. (DOCX 16 kb)

Additional file 5: Quality Appraisal Instrument for Qualitative Studies. (DOCX 258 kb)

Additional file 6: Quality Appraisal Instrument for Quantitative Studies. (DOCX 777 kb)
