# Supplementary material for: Digital storytelling as a method in health research: a systematic review protocol
Source: Syst Rev. 2018 Mar 5;7:41. doi: 10.1186/s13643-018-0704-y (PMC5838876; doi:10.1186/s13643-018-0704-y)
Supplement: Supplementary file 2 — MEDLINE (Ovid) Search Strategy. (DOCX 55 kb) [file 13643_2018_704_MOESM2_ESM.docx]

**Additional File 2: Medline (Ovid) Search Strategy**

1. (digital OR digitally) adj8 (stories OR story$).ab,kw,ti.
2. (digital OR digitally) adj3 (narrative$ OR dialogue$).ab,kw,ti.
3. visual$ adj3 (stories OR story$ OR narrative$ OR dialogue$).ab,kw,ti.
4. video$ adj (stories OR story$ OR diar$).ab,kw,ti.
5. (micro OR brief OR mini) adj movie$.ab,kw,ti.
6. 1 OR 2 OR 3 OR 4 OR 5
7. Narration/
8. Personal narratives as topic/
9. 7 OR 8
10. (digital$ OR multimedia).ab,kw,ti.
11. Video Recording/
12. Videotape Recording/
13. Multimedia/
14. 10 OR 11 OR 12 OR 13
15. 9 AND 14
16. 6 OR 15
17. Limit 16 to (English language and yr=”1990 –Current”)
