# Supplementary material for: Digital storytelling as a method in health research: a systematic review protocol
Source: Syst Rev. 2018 Mar 5;7:41. doi: 10.1186/s13643-018-0704-y (PMC5838876; doi:10.1186/s13643-018-0704-y)
Supplement: Supplementary file 3 — Verification of Study Eligibility Form. (DOCX 15 kb) [file 13643_2018_704_MOESM3_ESM.docx]

**Additional File 3: Verification of Study Eligibility Form**

| Author and year: |
| --- |
| Title: |
| Reviewer: |
| Population: pediatric or adult populations, their families, and/or health care professionals  Yes/No  Comments: |
| Phenomenon/intervention of interest: use of digital storytelling at any point in the research process  Yes/No  Comments: |
| Context: the research was conducted in healthcare settings (i.e. clinics, hospitals, community outreach, home visits) or by medical, nursing, or allied healthcare professionals. The research can take place in any geographical location.  Yes/No  Comments: |
| Study design: All quantitative and qualitative study designs will be included.  This study is original qualitative, quantitative, or mixed-methods research: Yes/No  The study is original qualitative research:  Qualitative design: _______________________  The study is original quantitative research:  Quantitative design: ______________________  The study is original mixed-methods research:  Quantitative design: ______________________  Comments: |
| The study was published between 1990 and (Date of Search): Yes/No |
| If you answer yes to all of the above questions, continue to quality assessment. If not, then exclude the paper.  Yes/No |
