# Supplementary material for: Digital storytelling as a method in health research: a systematic review protocol
Source: Syst Rev. 2018 Mar 5;7:41. doi: 10.1186/s13643-018-0704-y (PMC5838876; doi:10.1186/s13643-018-0704-y)
Supplement: Supplementary file 4 — Standardized Data Extraction Form. (DOCX 16 kb) [file 13643_2018_704_MOESM4_ESM.docx]

**Additional File 4: Standardized Data Extraction Form**

| Reviewer | | | Date | |
| --- | --- | --- | --- | --- |
| **Digital storytelling as a method for health research: A mixed-methods systematic review - Extraction Tool For Included Studies** | | | | |
| **Publication Information** | | | | |
| Title | | Author(s) | | |
| Date | Journal | | | |
| Country | | | | |
| **Theoretical Framework** | Done (Specify Which Theory) | | | Not Done |
| **Study Purpose, Design, and Methods** | | | | |
|  | | | | |
| **Context and Participants’ Characteristics** (i.e. clinical or community setting, geographical location, socio-demographic variables, and diagnosis) | | | | |
|  | | | | |
| **Participants’ Engagement and Role in the Research Process** | | | | |
|  | | | | |
| **Role and Training of the Researcher(s) Regarding Digital Storytelling** | | | | |
|  | | | | |
| **Digital Storytelling Purpose, Framework, and Processes** | | | | |
|  | | | | |
| **Ethical Considerations and Procedures Reported by Researcher(s)** | | | | |
|  | | | | |
| **Researchers’ and Participants’ Narrative Evaluation of Digital Storytelling** | | | | |
|  | | | | |
| **Findings of the Study** | | | | |
|  | | | | |
| **Study Conclusions** | | | | |
|  | | | | |
| **Knowledge Translation Initiatives** | | | | |
|  | | | | |
