# Supplementary figures and images for: Digital storytelling as a method in health research: a systematic review protocol
Source: Syst Rev. 2018 Mar 5;7:41. doi: 10.1186/s13643-018-0704-y (PMC5838876; doi:10.1186/s13643-018-0704-y)

**Additional File 5: Quality Appraisal Instrument for Qualitative Studies**


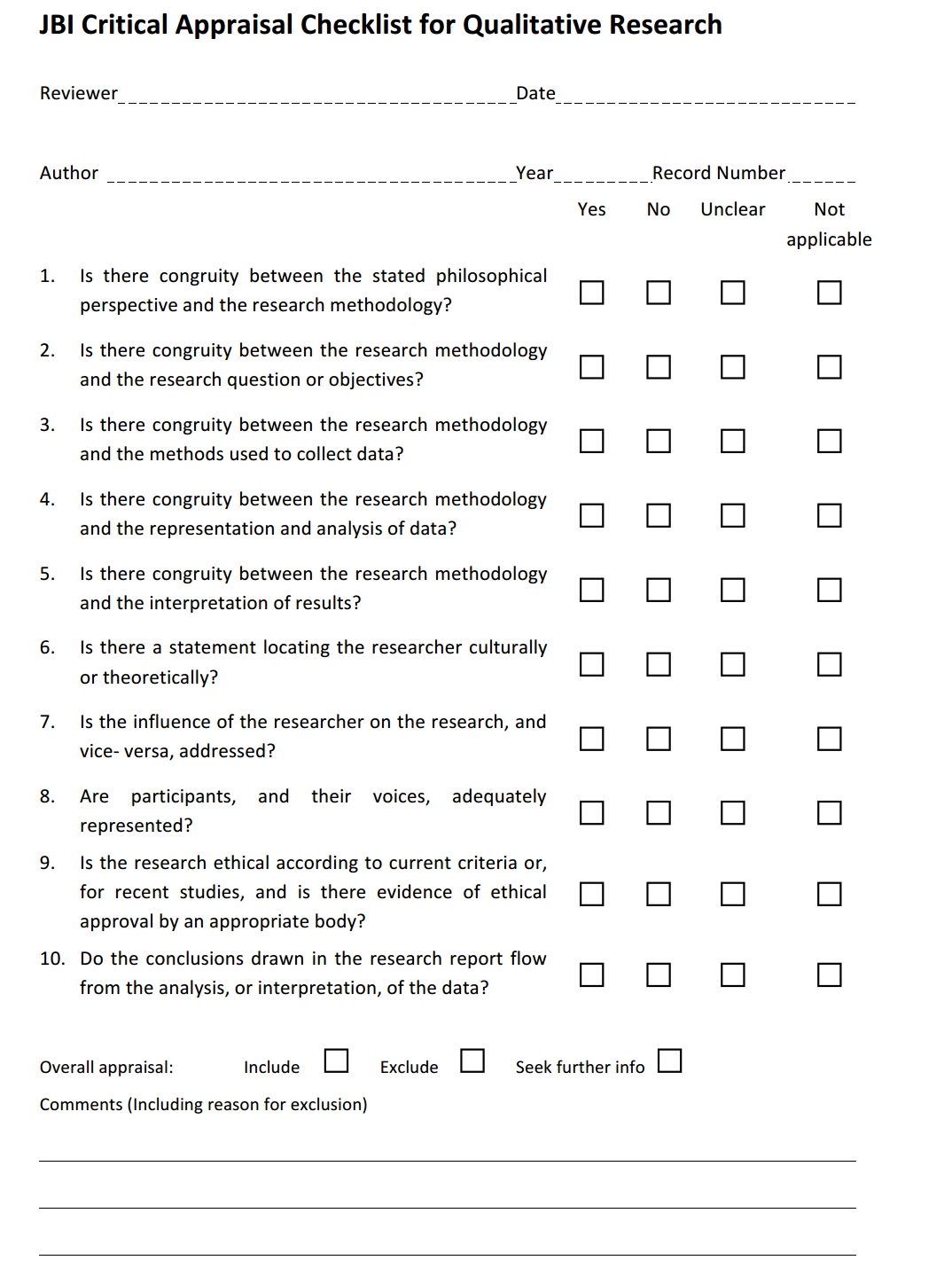

Supplement: Supplementary file 5 — Quality Appraisal Instrument for Qualitative Studies. (DOCX 258 kb) [file 13643_2018_704_MOESM5_ESM.docx]

**Additional File 6: Quality Appraisal Instrument for Quantitative Studies**

**
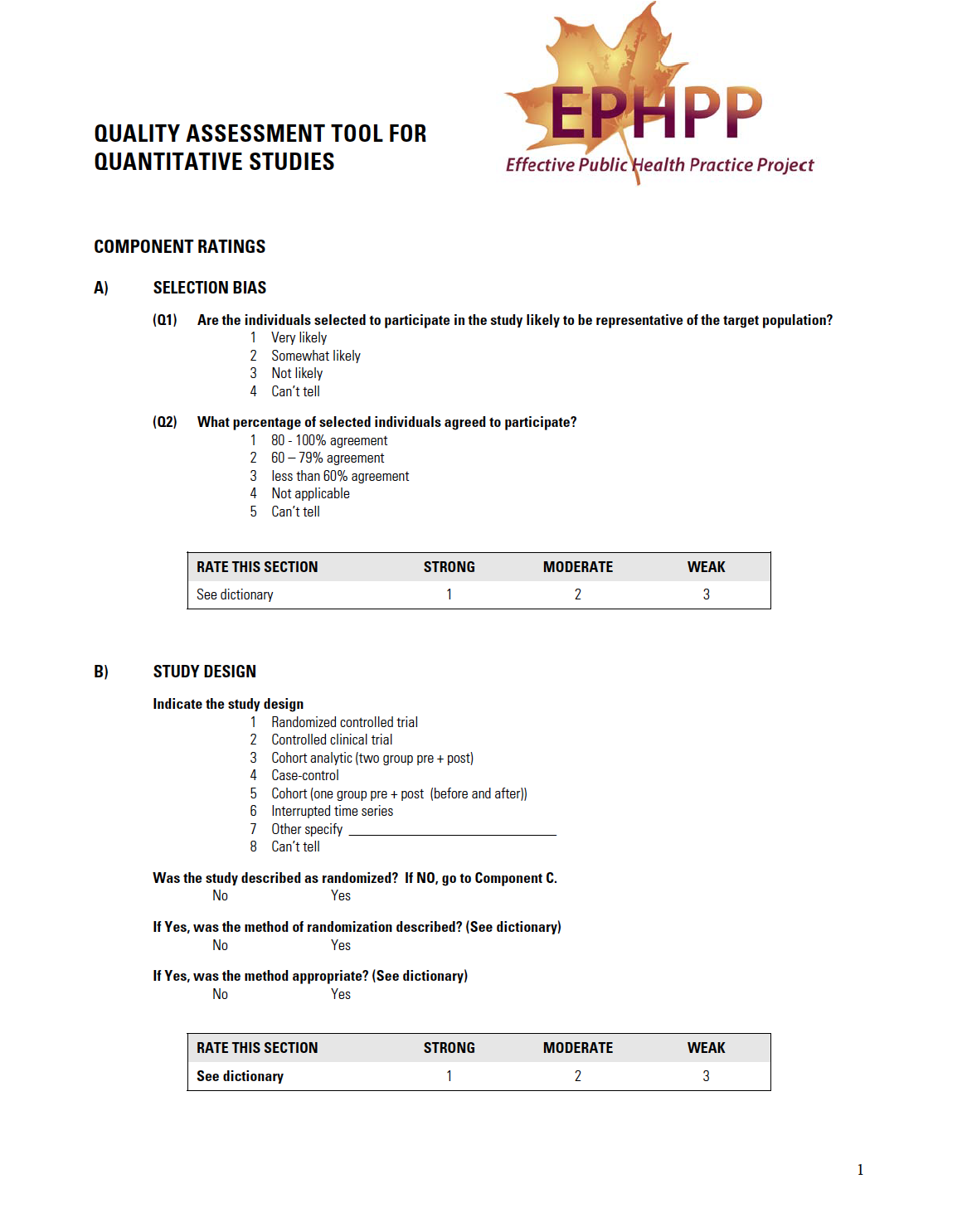
**

**
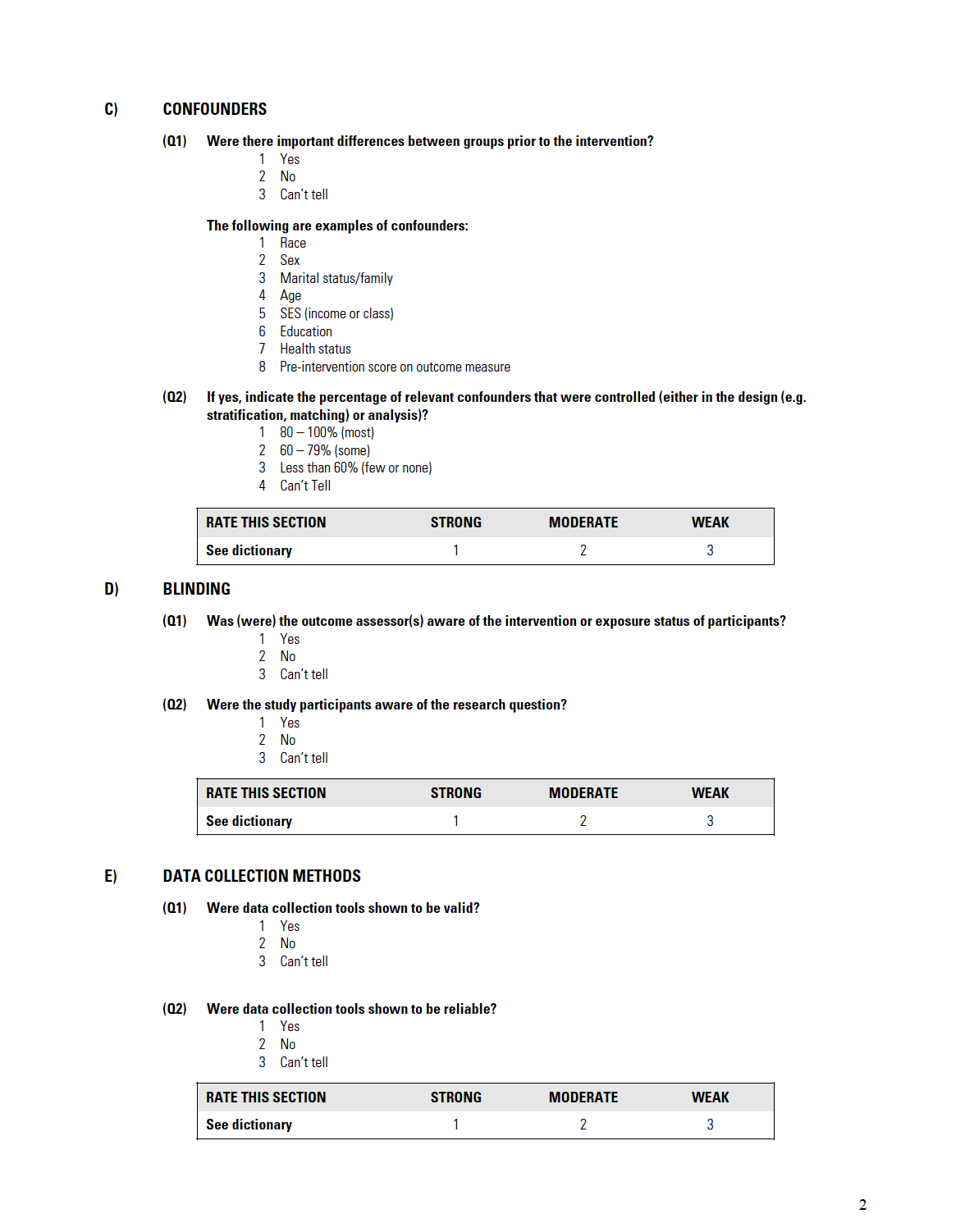
**

**
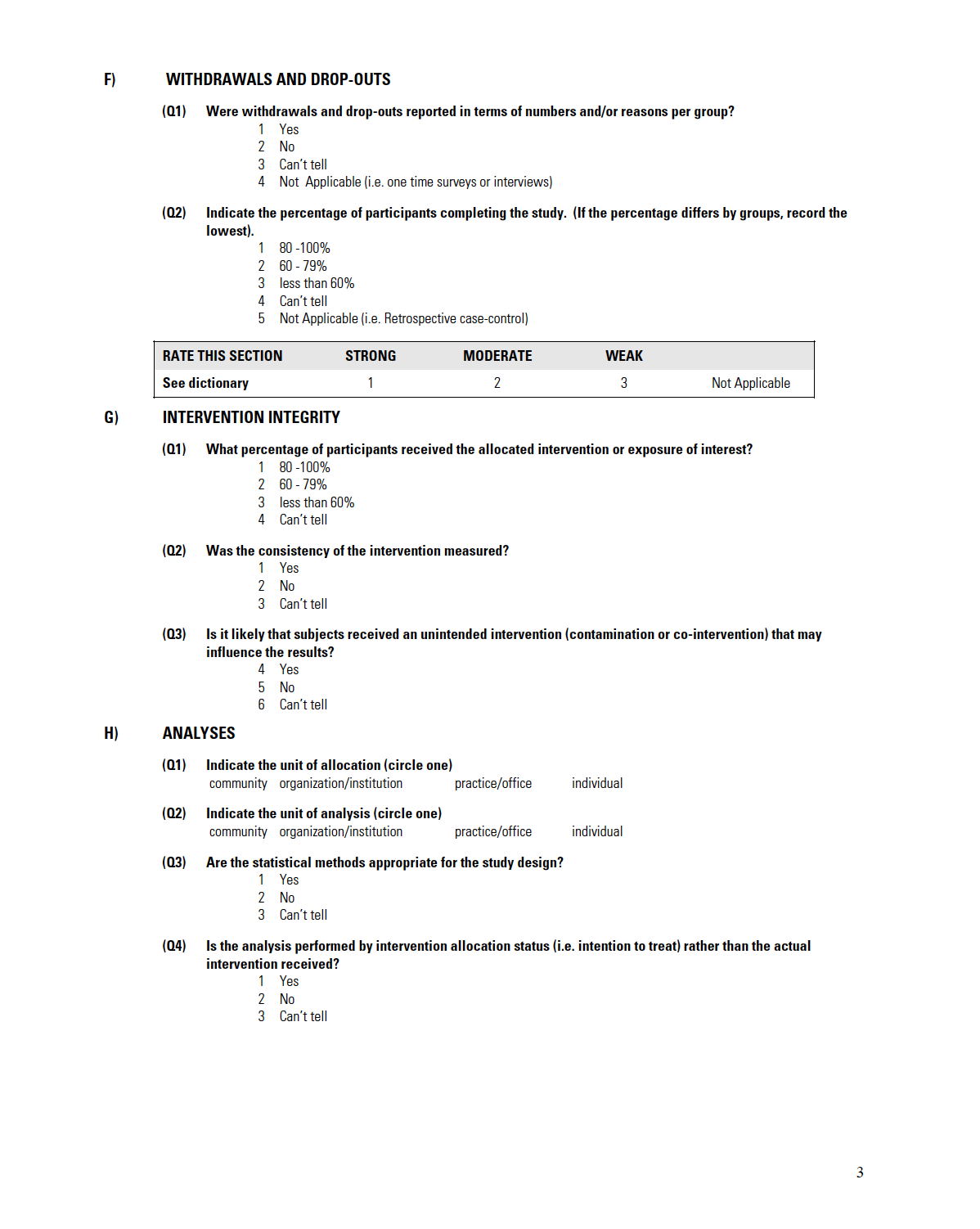
**

**
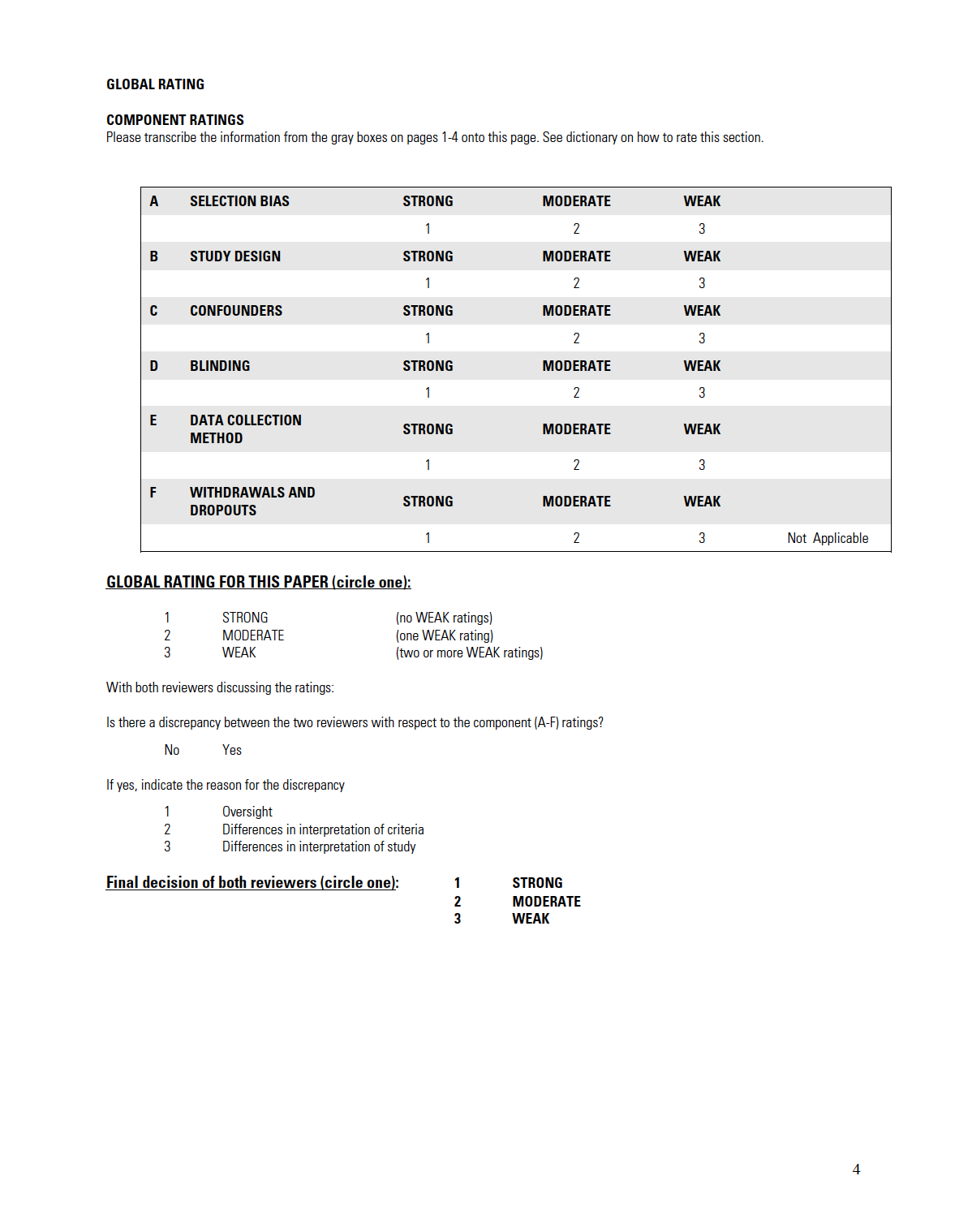
**

Supplement: Supplementary file 6 — Quality Appraisal Instrument for Quantitative Studies. (DOCX 777 kb) [file 13643_2018_704_MOESM6_ESM.docx]
